# Editorial: Cancer testis antigens in cancer: Recent developments as cancer biomarkers and therapeutic targets

**DOI:** 10.3389/fonc.2022.1075329

**Published:** 2022-11-10

**Authors:** Adviti Naik, Joe Yeong, Julie Decock

**Affiliations:** ^1^ Translational Cancer and Immunity Center, Qatar Biomedical Research Institute (QBRI), Hamad Bin Khalifa University (HBKU), Qatar Foundation (QF), Doha, Qatar; ^2^ Institute of Molecular and Cell Biology (ASTAR), Singapore, Singapore; ^3^ Division of pathology, Singapore General Hospital, Singapore, Singapore; ^4^ College of Health and Life Sciences (CHLS), Hamad Bin Khalifa University (HBKU), Qatar Foundation (QF), Doha, Qatar

**Keywords:** cancer testis antigen, cancer biology, cancer biomarker, cancer therapy, immunotherapy

Cancer testis antigens (CTAs) form a large family of proteins with highly restricted expression that is limited to male germ cells in the testis and trophoblast cells in the placenta. They are often re-expressed in tumors as a result of differential DNA methylation of their promoter regions, making them highly tumor-specific antigens. Moreover, CTAs are highly immunogenic as the immune system does not recognize them as self-proteins due to the immune privileged environment of the testis. Given their restricted expression patterns and immunogenic nature, CTAs have been identified as attractive candidate targets for anti-cancer therapy. This special issue was designed to highlight new advancements and insights into the oncogenic functions and biomarker or therapeutic potential of CTAs in cancer.

CTAs have been implicated in diverse aspects of oncogenesis where individual CTAs have been shown to increase genomic instability, promote tumor growth, invasion and metastasis, impede apoptosis, and enhance angiogenesis (1). Here, Traynor et al. demonstrate that Synovial Sarcoma, X-breakpoint (SSX) proteins are implicated in biological processes that regulate tumor growth as well as metastasis. More specifically, they show that silencing of overall SSX expression reduces tumor growth and completely inhibits metastatic burden of lung and liver *in vivo*. Molecularly, they found that *SSX* silencing induces cell cycle stalling, increased apoptosis and reduced migration and invasion potential of melanoma cells. Of note, using the TCGA repository they show that all six protein-coding SSX members are expressed in melanomas with *SSX1* and *SSX2* being expressed in almost 90% of primary melanomas and metastases, indicating that SSX proteins are attractive therapeutic targets for the majority of melanoma patients.

As compared to protein coding CTAs, to date, little is known about testis-specific lncRNAs and their role in cancer. One such lncRNA is Ret finger protein-like 3S (RFPL3S), an antisense transcript of the RFPL3 gene, that has been shown to act as a transcriptional factor on the human telomerase reverse transcriptase promoter. Guo et al. found that RFPL3S expression is downregulated in testicular germ cell tumors, negatively correlates with metastasis and is associated with higher disease-free interval and progression-free interval. Silencing RFPL3S significantly reduced tumor cell proliferation and invasion, concomitantly with gene expression changes in PI3K-Akt, Wnt and Hippo signaling as well as pathways related to focal adhesions, adherent junctions and extracellular matrix-receptor interactions. Interestingly, the authors also found that the expression of RFLP3S was positively correlated with immune infiltration of B cells, CD8+T cells, cytotoxic T cells, and NK cells, while the opposite was true in relation to infiltration of immunosuppressive cells such as Th17 and Th2. RFLP3S expression was also higher in patients who would likely benefit from immune checkpoint blockade as predicted by the Tumor Immune Dysfunction and Exclusion (TIDE) algorithm. These findings suggest that RFLP3S could be used as a prognostic biomarker and predictor of immunotherapy response in testicular germ cell cancer.

Similarly, Tu et al. report on the clinical significance of sperm autoantigen protein 17 (SPA17) as a predictive biomarker for response to immune checkpoint blockade. Pan-cancer analysis revealed that SPA17 expression is associated with poor prognosis in 15 cancers and good prognosis in 11, suggesting that SPA17 may exert multifaceted roles in a cancer-specific context. Gene set enrichment analysis showed that SPA17 expression is associated with differential expression of genes involved in epithelial-mesenchymal transition, immune-related pathways, inflammatory responses and immune cell infiltration across cancers. In addition, the authors found that high expression of SPA17 correlates with favorable response to PD-L1 treatment in urinary system tumors while the opposite was true in melanoma, further highlighting a likely tissue-specific role of SPA17 in cancer.

In turn, Bi et al. studied the immunotherapeutic potential of targeting the cancer testis antigen MAGE-D4 in glioma. The MAGE family has been widely studied in various cancers, and here the authors demonstrate that MAGE-D4 expression is upregulated in gliomas and is associated with poor prognosis. Using peptide binding prediction programs, peptide binding affinity and peptide/HLA-A2 stability assays they selected three native HLA-A2:0201 restricted peptides for further analysis and found that P8-pulsed dendritic cells were the strongest inducers of T cell proliferation, activation, and cytotoxicity. Treatment with epigenetic drugs enhanced the expression of MAGE-D4 and HLA-A2 and resulted in higher T cell cytotoxicity *in vitro* and reduced tumor burden *in vivo*, providing experimental evidence to support the potential use of MAGE-D4 immunotherapy in combination with epigenetic drugs for treatment of glioma.

In addition to using CTAs as immunotherapeutic targets, their immunogenic nature also lends them to being used as biomarkers of the anti-tumor immune response. In this context, Miyamoto et al. investigated the potential biomarker value of monitoring CTA autoantibodies to predict clinical outcome. They designed a multiple S-cationized antigen-immobilized bead array (MUSCAT) assay system that enables the quantification of polyclonal antibodies against linear CTA epitopes and can be used to assess antigen spreading. As a proof-of-concept, they used the MUSCAT system to monitor CTA autoantibody levels in serum samples from patients with metastatic castration-resistant prostate cancer who received adenovirus−mediated REIC/Dkk−3 (Ad-REIC) gene therapy. As expected, Ad-REIC treatment induced tumor cell apoptosis, resulting in the activation of the anti-tumor immune response as detected by the MUSCAT assay which showed higher levels of CTA-specific autoantibodies and antigen spreading during treatment. Of note, in accordance with tumor regression CTA autoantibody levels decreased and preceded changes in PSA levels suggesting that the MUSCAT assay could be used to monitor treatment immune responses and disease burden.

Within the area of CTA biomarkers, the study by Peng et al. focuses on the biomarker value of LDHC in serum and exosomes of lung adenocarcinoma patients. They demonstrate that *LDHC* expression in serum and serum-derived exosomes is elevated in patients with lung adenocarcinoma and increases with disease progression. Furthermore, they show that exosomal *LDHC* expression could be used to monitor treatment efficacy and disease recurrence.

Finally, Yang et al. review the recent advancements in the field on the role of CTAs in lung cancer development and metastasis, discuss their potential as biomarkers and therapeutic targets and provide some future perspectives for CTA-targeted cancer therapy. The authors highlight some of the key challenges that face CTA-based immunotherapy such as the lack of cell-surface CTA expression for CAR-T cell-based therapy and defects in MHC class I antigen presentation that prevent cell-surface presentation of intracellular CTAs. Further, they discuss few opportunities for further development including genetic and epigenetic editing to knock out or turn off oncogenic CTAs, or to turn on tumor suppressive CTAs.

To conclude, this collection of articles illustrates how CTAs contribute to tumorigenesis and tumor progression and could be used in clinical practice as biomarkers of disease, treatment response and anti-tumor immunity. This Research Topic also sheds some light on emerging therapeutic opportunities for CTA-based cancer treatment.

## Author contributions

AN, JY and JD contributed to writing the manuscript. All the authors proof read the manuscript and approved the submitted version.

## Funding

This work was supported by a grant from the Qatar Biomedical Research Institute (#VR94), awarded to JD and funding from the A*STAR Biomedical Engineer Programme (#C211318003) and Singapore National Medical Research Council (#MOH-000323-00, #OFYIRG19may-0007) awarded to JY.

## Conflict of interest

The authors declare that the research was conducted in the absence of any commercial or financial relationships that could be construed as a potential conflict of interest.

## Publisher’s note

All claims expressed in this article are solely those of the authors and do not necessarily represent those of their affiliated organizations, or those of the publisher, the editors and the reviewers. Any product that may be evaluated in this article, or claim that may be made by its manufacturer, is not guaranteed or endorsed by the publisher.
